# Suppression of Quorum Sensing and Virulence Factors by Meloxicam and Celecoxib in Methicillin-Resistant *Staphylococcus aureus* Clinical Isolates

**DOI:** 10.3390/antibiotics15070707

**Published:** 2026-07-21

**Authors:** Reham Ali, Ramadan A. El-Domany, Mona I. Shaaban

**Affiliations:** 1 Department of Microbiology and Immunology, Faculty of Pharmacy, Kafrelsheikh University, Kafr El Sheikh 33516, Egypt; reham_mansour@pharm.kfs.edu.eg (R.A.);; 2 Department of Microbiology and Immunology, Faculty of Pharmacy, Mansoura University, Mansoura 35516, Egypt

**Keywords:** *Staphylococcus aureus*, meloxicam, celecoxib, NSAIDs, quorum sensing, virulence, docking, hemolysin, protease, lipase

## Abstract

**Background:** *Staphylococcus aureus* is a major cause of life-threatening infections, and increasing antimicrobial resistance necessitates alternative therapeutic strategies. This study evaluated the antiquorum sensing and antivirulence activities of selected nonsteroidal anti-inflammatory drugs (NSAIDs) against *S. aureus* clinical isolates by targeting the accessory gene regulator (*agr*). **Methods:** Antimicrobial susceptibility and virulence factor production were evaluated in 56 *S. aureus* clinical isolates. The MICs of six NSAIDs (Diclofenac, ketoprofen, ketorolac, meloxicam, indomethacin and celecoxib) were determined by broth microdilution, and the effects of sub-MICs (½ and ¼ MIC) on virulence factors were assessed. The expression of *agrA*, *hlb*, and *hld* was analyzed by qRT-PCR, and molecular docking was performed to evaluate interactions with AgrA receptor. **Results:** Among the isolates, high resistance rates were accompanied with cefoxitin, ceftazidime, and cefepime. Hemolysin, protease, and lipase production were detected in 42.86%, 89.28%, and 94.64% of isolates, respectively. Meloxicam and celecoxib at sub-MIC levels significantly reduced hemolysin, protease, and lipase activities. Additionally, both drugs markedly downregulated *agrA* expression by 88.9–95.3% and significantly reduced *hlb* and *hld* expression by 90.5–99.9% without affecting bacterial growth. Molecular docking demonstrated favorable binding interactions with AgrA. **Conclusions:** Meloxicam and celecoxib could be promising adjunctive therapies against *S. aureus* through suppression of the Agr-regulated virulence traits.

## 1. Introduction

*Staphylococcus aureus* is a Gram-positive, facultative anaerobic bacterium that is a major cause of both community acquired and healthcare associated infections worldwide [1]. It is capable of colonizing the skin, and mucous membranes of humans, and shifting from a commensal state to a highly virulent pathogen under certain conditions. *S. aureus* is implicated in a diverse range of clinical diseases, including superficial skin and soft tissue infections. Common diseases include impetigo, folliculitis, cellulitis, and abscesses, while more invasive diseases involve pneumonia, osteomyelitis, septic arthritis, bacteremia, endocarditis, and device-associated infections. The organism’s ability to secrete potent exotoxins, such as toxic shock syndrome toxins and exfoliative toxins, contributes to disease severity and persistence [2,3,4].

Antimicrobial resistance in *S. aureus*, particularly among methicillin-resistant *S. aureus* (MRSA), continues to limit therapeutic options and poses a major global health challenge [5,6,7]. In addition to antibiotic resistance, the pathogenicity of *S. aureus* is strongly influenced by virulence regulation [8], highlighting the need for alternative therapeutic strategies that target bacterial virulence rather than viability [9,10].

Quorum sensing (QS) in *S. aureus* is a complicated cell-to-cell communication mechanism that regulates the expression of virulence factors, biofilm formation, and colonization behaviors in response to bacterial population density [11]. The primary QS system in *S. aureus* is the accessory gene regulator (Agr) system, which functions as a global regulator of virulence gene expression and modulates the pathogen’s transition between colonization and invasive infection [12].

The central QS system consists of two divergent promoters, P2 and P3, driving transcription of RNAII and RNAIII, respectively. RNAII encodes the structural components AgrB, AgrD, AgrC, and AgrA, which mediate autoinducing peptide (AIP) synthesis, processing, secretion, and detection. AgrD serves as the pro-peptide precursor of AIP, while AgrB processes and exports it. Upon accumulation to the threshold concentration of AIP, extracellular AIP binds and activates the membrane bound histidine kinase AgrC, which in turn phosphorylates the response regulator AgrA [13]. Activated AgrA promotes further expression of RNAII, amplifying the system, and induces transcription of RNAIII, the major effector molecule. RNAIII, together with AgrA, orchestrates a global regulatory switch, upregulating genes encoding secreted toxins and enzymes while downregulating surface adhesins. This regulation ensures that *S. aureus* initially adheres to host tissues and subsequently produces invasive factors to disseminate, highlighting the Agr QS system as a vital determinant of pathogenesis and a potential therapeutic target [11,14].

Some drugs have gained attention as a repurposed antivirulence agent due to its ability to interfere with *agr*-mediated regulatory events in *S. aureus*. Sitagliptin, a novel antidiabetic agent, significantly downregulated the *S. aureus* QS gene *agrA*, the global virulence regulators *sarA* and *sigB*, as well as biofilm-associated genes *icaA* and *fnbA* [15]. Also, the diuretic agent bumetanide was effectively repurposed and demonstrated pronounced antivirulence activity in *S. aureus* via inhibition of the AgrA QS regulator [16]. Several nonsteroidal anti-inflammatory drugs (NSAIDs) exhibit antivirulence activity by disrupting QS and biofilm formation. Diflunisal suppresses *agr*-regulated toxins in *S. aureus* [17], meloxicam and diclofenac attenuate *Pseudomonas aeruginosa* virulence [18,19], and diclofenac and ketorolac inhibit urease activity and biofilm formation in uropathogenic *Proteus mirabilis* [20]. In addition, meloxicam-curcumin and diclofenac-curcumin also showed synergistic antibacterial effects against *S. aureus* and *Enterococcus faecium* [21]. Celecoxib also exhibits antibacterial activity against MRSA and acts synergistically with conventional antibiotics [22]. However, the anti-QS and antivirulence activity of other NSAIDs have not been fully studied in the *S. aureus* Agr system.

In this context, the present study aimed to evaluate the effects of some selected NSAIDs on the Agr QS system and its associated virulence factors, including hemolysin, protease, and lipase, in clinical isolates of *S. aureus*. The influence of the most promising inhibitors on *agr*-mediated virulence was assessed phenotypically and further validated using quantitative real-time PCR and molecular docking analyses.

## 2. Results

### 2.1. Source and Identification of S. aureus Clinical Isolates

**Figure 1:**
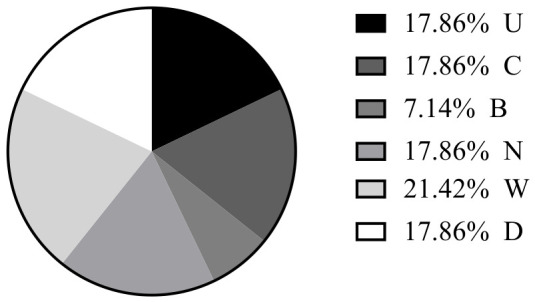
Clinical sources of isolates; U: urine, C: conjunctivitis, B: blood, N: nasal swaps, W: wound, D: diabetic foot.

In this study, a total of 56 *S. aureus* isolates were obtained from various clinical sources, including 10 from urine, 10 from conjunctivitis patients, four samples from blood, 10 from nasal swaps, 12 from wounds and 10 diabetic foot cases. Under light microscopy, the isolates were Gram-positive cocci with grape like clusters (Supplementary Figure S1). After incubation at 37 °C for 24 h on nutrient agar and mannitol salt agar plates, the colonies were characterized with round shape and golden-yellow color. Sources of the *S. aureus* samples are shown in Figure 1 and Supplementary Table S1. All the isolates were coagulase positive. Among the 56 isolates examined, clot formation was observed in 21.42% after 1 h of incubation, 50% after 2 h, 8.92% after 3 h, 1.79% after 4 h, 3.57% after 5 h, 1.79% after 6 h, and 12.5% following overnight incubation. These findings indicate that the majority of the isolates exhibited strong coagulase activity within the initial 2 h of incubation.

### 2.2. Determination of Antibiotic Susceptibility to Different Classes of Antimicrobial Agents

#### 2.2.1. The Antibiotic Susceptibility of *S. aureus* Isolates

**Figure 2:**
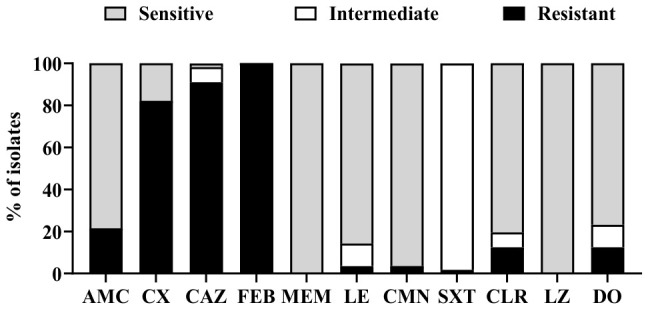
Prevalence of *S. aureus* resistance to different antimicrobial agents.

All *S. aureus* isolates exhibited complete resistance to cefepime (100%), whereas they were susceptible to linezolid and meropenem (100%). In contrast, varying degrees of resistance were observed against the remaining nine antimicrobials. Specifically, resistance was detected in 12 isolates (21.43%) to amoxicillin/clavulanic acid, 46 isolates (82.14%) to cefoxitin, 51 isolates (91.07%) to ceftazidime. Lower resistance rates were recorded for sulfamethoxazole/trimethoprim (1 isolate, 1.78%), levofloxacin (2 isolates, 3.57%), clindamycin (2 isolates, 3.57%), both clarithromycin, and doxycycline (7 isolates, 12.5%) (Figure 2).

The distribution of antimicrobial resistance according to the clinical source is summarized in the Supplementary Materials (Supplementary Figure S2) and was included to characterize the diversity of the clinical isolate collection.

#### 2.2.2. The Antibiotic Susceptibility of *S. aureus* Isolates to Vancomycin

**Figure 3:**
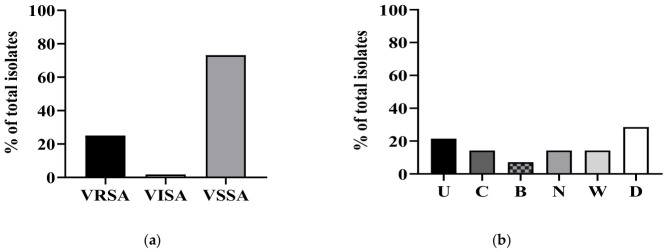
Determination of vancomycin susceptibility among *S. aureus* isolates. (**a**) Detection of VRSA, VISA and VSSA among *S. aureus* isolates. (**b**) Distribution of VRSA among different clinical sources of *S. aureus* (urine, conjunctivitis, blood, nose, wound, diabetic foot). VRSA: vancomycin-resistant *S. aureus*, VISA: vancomycin intermediate *S. aureus*, VSSA: vancomycin sensitive *S. aureus*, U: urine, C: conjunctivitis, B: blood, N: nasal swaps, W: wound, D: diabetic foot.

Based on the CLSI MIC breakpoints, 25% of the isolates could be classified as VRSA (MIC ≥ 16 µg/mL), while 1.79% could be classified as VISA (MIC 4–8 µg/mL). The highest proportion of VRSA was detected in isolates from diabetic foot infections (28.57%), followed by urine (21.43%), wound isolates (14.29%), nasal swabs (14.29%), conjunctivitis samples (14.29%) and blood (7.14%). VISA isolate was also detected in isolates no. 6 SA, which was obtained from urine (Figure 3 and Supplementary Table S2).

### 2.3. Determination of Different Virulence Factors in the Tested Isolates

#### 2.3.1. Hemolysis Assay

**Figure 4:**
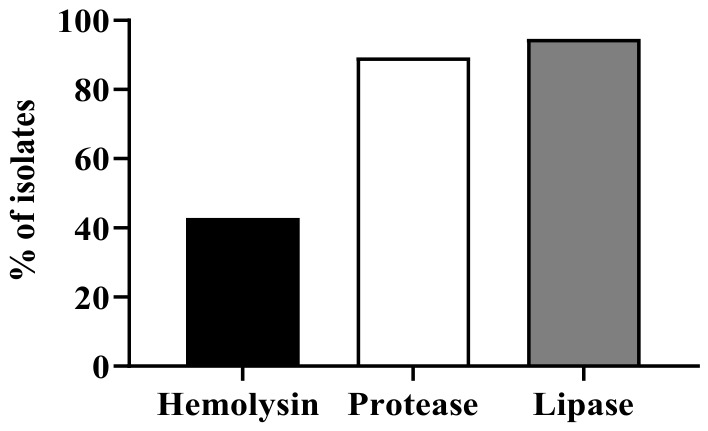
Prevalence of virulence factors (hemolysin, protease and lipase) among *S. aureus* tested isolates. Statistical significance was determined using one-way ANOVA followed by Dunnett’s multiple comparison test.

The hemolytic activity of *S. aureus* isolates was assessed to evaluate their potential virulence performance. Among the 56 *S. aureus* clinical isolates, 42.86% caused significant hemolysis of RBCs (*n* = 24/56) (*p* < 0.05) (Figure 4). Hemolysis was also detected across various clinical sources, with 25% from urine (*n* = 6/24), 20.83% from conjunctivitis samples (*n* = 5/24), 4.17% from blood (*n* = 1/24), 8.33% from nasal swabs (*n* = 2/24), 16.67% of isolates from wounds (*n* = 4/24) and 25% from diabetic foot infections (*n* = 6/24).

#### 2.3.2. Protease Assay

Protease production among *S. aureus* isolates was evaluated via the use of 5% skim milk as a substrate. Overall, 89.28% of the isolates exhibited protease activity (*n* = 50/56), with a statistically significant difference (*p* < 0.05) compared to the negative control (Figure 4). Further analysis across different clinical sources revealed that 16% of the isolates from urine (*n* = 8/50), 18% of the isolates from conjunctivitis samples (*n* = 9/50), 6% of isolates derived from blood (*n* = 3/50), and 20% from the nasal swabs were protease producers (*n* = 10/50). Additionally, 22% from wounds (*n* = 11/50) and 18% from diabetic foot infections (*n* = 9/50) demonstrated proteolytic activity.

#### 2.3.3. Lipase Assay

Lipase activity was assessed using para-nitrophenyl palmitate (pNPP) as a substrate, and enzymatic activity was quantified by measuring the hourly release of para-nitrophenol (pNP) relative to a negative control. Among the 56 *S. aureus* isolates tested, 94.64% exhibited lipase production (*n* = 53/56), indicating a high prevalence of this virulence factor (Figure 4). When stratified by clinical source, 16.98% of the isolates from urine samples (*n* = 9/53), 18.87% conjunctivitis samples (*n* = 10/53), 7.55% from blood (*n* = 4/53), and 18.87% from nasal swabs from healthcare personnel (*n* = 10/53) were lipase producers. Moreover, 18.87% from wounds (*n* = 10/53) and 18.87% from diabetic foot infections (*n* = 10/53) were positive for lipase production.

### 2.4. Detection of Quorum Sensing and Associated Virulence in Some Selected Isolates

#### 2.4.1. Criteria for Isolates Selection

Isolates 15SA, 25SA, and 47SA were selected for further evaluation based on their virulence characteristics and antimicrobial resistance profiles. Isolates 25SA and 47SA exhibited hemolytic, proteolytic, and lipolytic activities, whereas isolate 15SA demonstrated hemolytic and proteolytic activities. In addition, isolates 15SA and 47SA were identified as MRSA, while isolate 25SA was identified as MRSA/VRSA. Based on these phenotypic characteristics, the selected isolates were used to evaluate the antivirulence effects of the tested NSAIDs.

#### 2.4.2. Detection of Quorum Sensing and Associated Virulence Genes in Some Selected Isolates by PCR

All targeted QS and virulence-associated genes were amplified from the selected isolates, yielding PCR products appearing at the expected fragment sizes, consistent with the expected amplicon lengths (Supplementary Table S3).

The QS related gene (*agrA*) was identified in the selected isolates. Additionally, several key virulence genes were detected including those encoding alpha-, beta- and delta-hemolysins (*hla*, *hlb* and *hld*), Staphylococcal protein A (*spa*), and phenol-soluble modulins (*psm*). In contrast, the toxic shock syndrome toxin (*tst*) was detected only in isolate 47SA. Meanwhile, the lipase gene (*hub*) and exfoliative toxin A gene (*eta*) were present in isolates 25SA and 47SA.

### 2.5. Minimum Inhibitory Concentrations (MICs) of the Selected Drugs

First, the MIC of each of the selected drugs was determined against *S. aureus* isolates (15SA, 25SA and 47SA). Then, sub-MIC concentrations of the drugs were subsequently tested for their ability to modulate *agr*-related virulence factors, including hemolysin, protease and lipase. The MICs of diclofenac against *S. aureus* isolates 15SA, 25SA and 47SA ranged from 0.156 to 2.5 mg/mL, while the MICs of ketoprofen against the same isolates ranged from 2.5 to 5 mg/mL. Ketorolac MICs ranged from 1.25 to 2.5 mg/mL. The range of meloxicam MICs was 0.625 to 2.5 mg/mL, whereas the MIC range of indomethacin was 0.312 to 2.5 mg/mL. As well, the MICs of celecoxib against these isolates were from 1.25 to 5 mg/mL. The MICs of the selected drugs are recorded in Table 1. To evaluate the impact of the selected drugs on *agr*-mediated pathogenicity, they were tested at sub-MIC concentrations (¼ and ½ MIC) for their inhibitory effects on QS and virulence determinants.

### 2.6. The Effect of Sub-MICs on Bacterial Growth

At multiple time intervals, the OD_600nm_ of treated and untreated cultures was recorded to assess bacterial growth kinetics. The ODs of *S. aureus* were estimated after treatment with ½ MIC of the selected drugs (diclofenac, ketoprofen, ketorolac, meloxicam, indomethacin and celecoxib).

**Figure 5:**
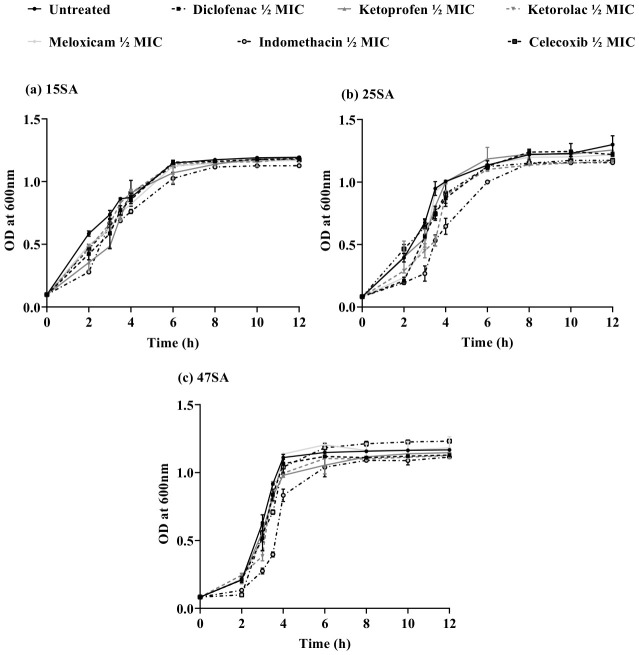
Growth curve of *S. aureus* isolates in the absence and presence of ½ MIC of the tested drugs. (diclofenac, ketoprofen, ketorolac, meloxicam, indomethacin and celecoxib). (**a**) Isolate no. 15SA treated with the tested NSAIDs. (**b**) Isolate no. 25SA treated with the tested NSAIDs (**c**) Isolate no. 47SA treated with the tested NSAIDs. Data are presented as the mean ± SD. Statistical analysis was performed using two-way repeated-measures ANOVA followed by Sidac’s multiple comparisons test. *p* < 0.05.

Treatment with ½ MIC of diclofenac, ketoprofen, ketorolac, meloxicam, indomethacin, and celecoxib did not markedly affect the growth kinetics of the tested *S. aureus* isolates (15SA, 25SA, and 47SA) compared with the untreated controls (Figure 5a–c). Treated cultures with diclofenac, ketoprofen, meloxicam, and celecoxib reached the exponential phase within 3.5 h, with OD_600nm_ values ranging from 0.745–0.805, 0.715–0.83, 0.786–0.92, and 0.697–0.74, respectively. Ketorolac-treated cultures entered the exponential phase within 3.5 h for isolates 15SA and 47SA (OD_600nm_ = 0.83–0.92) and after 4 h for isolate 25SA (OD_600nm_ = 0.89). Indomethacin slightly delayed bacterial growth, with all isolates reaching the exponential phase within 4–5 h and OD_600nm_ values ranging from 0.76–0.832. Overall, the growth profiles remained comparable to those of the untreated controls.

### 2.7. The Effect of Sub-MICs on Virulence Factors

#### 2.7.1. Hemolysis Activity

First, hemolysin production by *S. aureus* isolates (15SA, 25SA and 47SA) in response to ½ and ¼ MICs of NSAID was assessed by measuring hemoglobin release from lysed RBCs. The findings demonstrated that *S. aureus* isolates no. 15SA, 25SA and 47SA exposed to ½ MIC of diclofenac presented significant decreases in hemolysin production by 49.24%, 36.75% and 83.33%, respectively (*p* < 0.05). At low level, diclofenac (¼ MIC) significantly decreased hemolysin production in *S. aureus* isolates no. 15SA by 48.63%. Similarly, ketorolac at ½ MIC and ¼ MIC significantly decreased hemolysin production in *S. aureus* isolate no. 15SA by 31.95% and 19.61%, respectively. Likewise, hemolysin production in *S. aureus* isolate no. 47SA was significantly decreased by 19.85% (*p* < 0.05).

**Figure 6:**
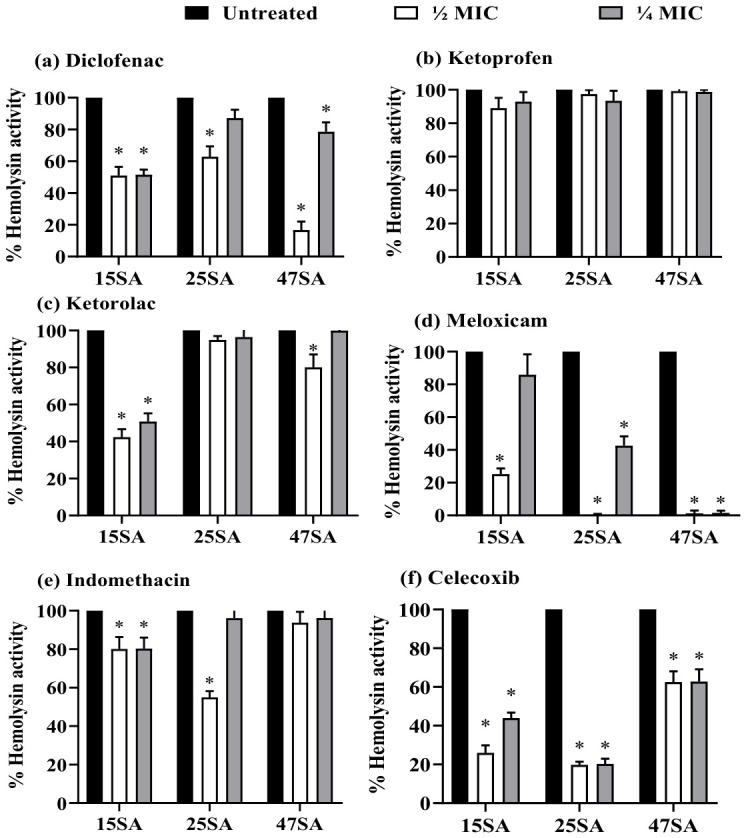
Effects of sub-MICs (½ and ¼ MICs) of NSAIDs on hemolysin production in *S. aureus* isolates (15SA, 25SA and 47SA). (**a**) Treatment with diclofenac, (**b**) ketoprofen, (**c**) ketorolac, (**d**) meloxicam, (**e**) indomethacin, and (**f**) celecoxib. Each experiment was conducted in quadruplicate, and the results are expressed as the means ± SDs. Statistical analysis was performed using paired *t* test. * *p* < 0.05.

By comparison, the effect of meloxicam was prominent. Meloxicam at ½ MIC significantly decreased the hemolysin production in *S. aureus* isolates (15SA, 25SA and 47SA) by 74.88%, 99.32% and 98.65%, respectively. In the same way, meloxicam at ¼ MIC significantly decreased the hemolysis production in *S. aureus* isolates (15SA, 25SA and 47SA) by 14.88%, 56.96% and 98.38%, respectively (*p* < 0.05). Also, hemolysin production in *S. aureus* isolate no. 15SA was significantly decrease by 20.19% and 20.03% when this isolate was treated with ½ and ¼ MIC of indomethacin, respectively. Similarly, indomethacin at ½ MIC exhibited a significant decrease in hemolysin production by 44.7% in *S. aureus* isolate 25SA. However, indomethacin (½ and ¼ MIC) showed less effect on isolate 47SA. As well, celecoxib exhibited promising results. Celecoxib at ½ MIC showed a significant decrease in hemolysin production in *S. aureus* isolates 15SA, 25SA and 47SA by 73.85%, 79.64% and 37.49%, respectively. In the same way, treatment of *S. aureus* isolates 15SA, 25SA and 47SA with ¼ MIC of celecoxib demonstrated a significant decrease in hemolysin production by 55.91%, 79.22% and 37.49%, respectively (Figure 6).

#### 2.7.2. Protease Assay

Protease activity was evaluated by determining the absorbance of culture supernatants from drug treated samples and comparing the results with those of untreated cultures. Diclofenac at ½ MIC significantly decrease protease activity by 17.7%, 76.19% and 82.6% in *S. aureus* isolates no. 15SA, 25SA and 47SA, respectively, while treatment of *S. aureus* isolates no. 25SA and 47SA with ¼ MIC of diclofenac presented significant decreases in protease activity by 57.19% and 75.58%, respectively (*p* < 0.05). In addition, treatment of *S. aureus* isolates no. 15SA, 25SA and 47SA with ½ MIC of ketoprofen significantly decreased protease production by 77.5%, 98.94% and 99.49%, respectively (*p* < 0.05). However, ketoprofen at ¼ MIC significantly decreased protease activity by 73.8% and 52% in *S. aureus* isolates no. 15SA and 25SA, respectively. Ketorolac at ½ MIC significantly decreased protease activity by 55.7%, 18.98% and 99.07% in *S. aureus* isolates no. 15SA, 25SA and 47SA, respectively, whereas at ¼ MIC, ketorolac significantly decreased protease activity in *S. aureus* isolates no. 15SA and 47SA by 54.5% and 25.09%, respectively.

**Figure 7:**
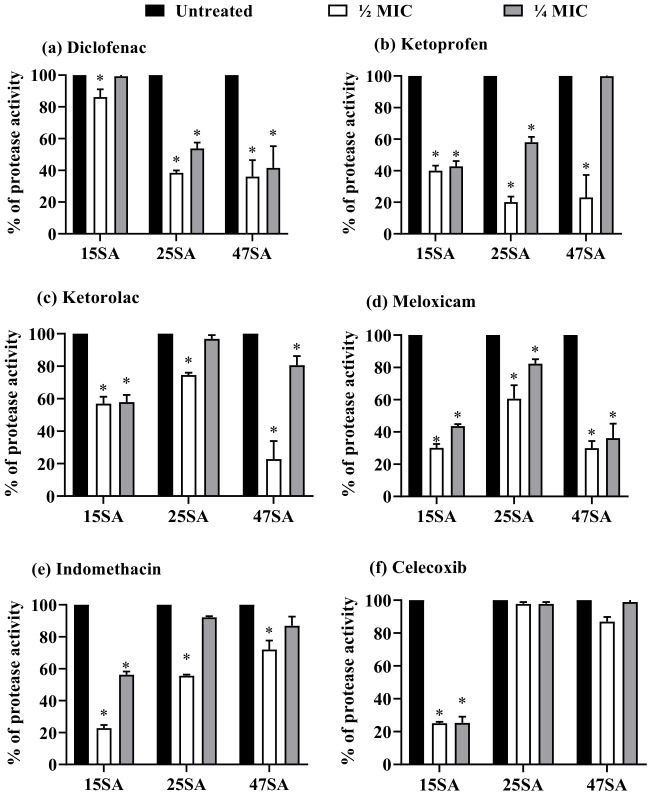
Effects of sub-MICs (½ and ¼ MICs) of NSAIDs on protease production in *S. aureus* isolates (15SA, 25SA and 47SA). (**a**) Treatment with diclofenac, (**b**) ketoprofen, (**c**) ketorolac, (**d**) meloxicam, (**e**) indomethacin, and (**f**) celecoxib. Each experiment was conducted in quadruplicate, and the results are expressed as the means ± SDs. Statistical analysis was performed using paired *t* test. * *p* < 0.05.

Meloxicam illustrated significant decrease in protease activity in all *S. aureus* isolates. It showed significant decrease at ½ MIC in isolates no. 15SA, 25SA and 47SA by 90.05%, 48.86% and 90.34%, respectively. As well, at ¼ MIC, it decreased protease activity significantly by 72.7%, 22.04% and 81.99% in isolates no. 15SA, 25SA and 47SA, respectively. Additionally, indomethacin at ½ MIC significantly decreased protease activity in isolates no. 15SA, 25SA and 47SA by 99.65%, 55.07% and 36.08%, respectively. At ¼ MIC, indomethacin significantly decreased protease activity by 56.57% in isolate no. 15SA and showed low effect on isolates 25SA and 47SA. Moreover, the protease activity of *S. aureus* isolate no. 15SA decreased significantly by 96.6% and 96.43% when this isolate was treated with ½ and ¼ MIC of celecoxib, respectively (Figure 7).

#### 2.7.3. Lipase Assay

The impact of NSAIDs at ¼ and ½ MIC on lipase activity in *S. aureus* isolates 25SA and 47SA was quantitatively evaluated by measuring para-nitrophenol (p-NP) release. Isolate 15SA was not included in the lipase assay, as PCR analysis did not detect the lipase gene. Diclofenac at ½ MIC significantly decrease p-NP concentration by 62.88% and 29.01% in *S. aureus* isolates no. 25SA and 475SA, respectively. However, treatment of *S. aureus* isolate no. 25SA with ¼ MIC of diclofenac presented significant decreases in p-NP concentrations by 43.22% (*p* < 0.05).

**Figure 8:**
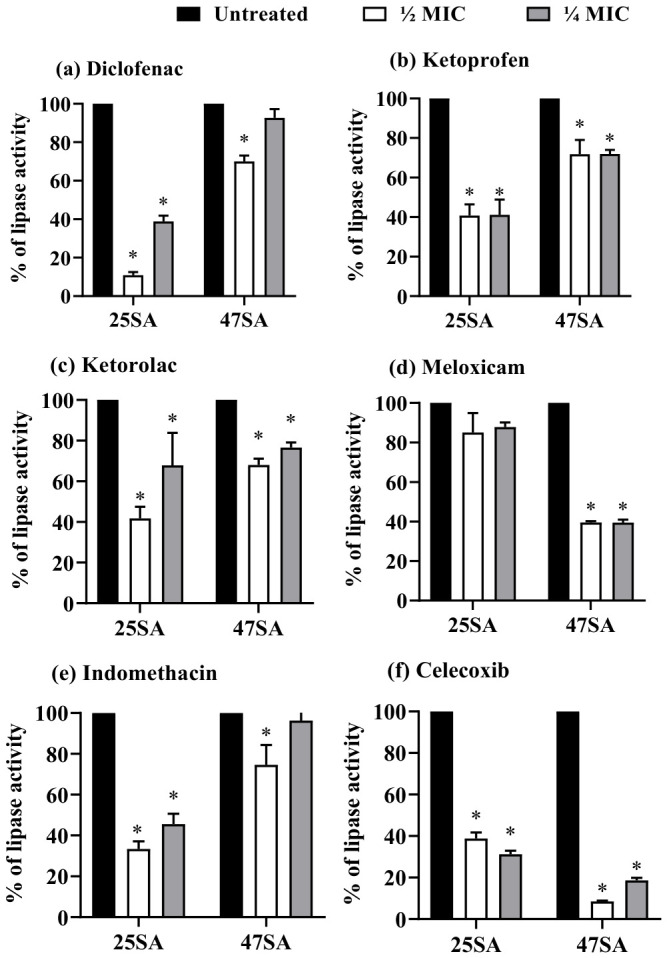
Effects of sub-MICs (½ and ¼ MICs) of the tested drugs on lipase activity in *S. aureus* isolates (25SA and 47SA). (**a**) Treatment with diclofenac, (**b**) ketoprofen, (**c**) ketorolac, (**d**) meloxicam, (**e**) indomethacin, and (**f**) celecoxib. Each experiment was conducted in quadruplicate, and the results are expressed as the means ± SDs. Statistical analysis was performed using paired *t* test. * *p* < 0.05.

In addition, treatment of *S. aureus* isolates no. 25SA and 47SA with ½ MIC of ketoprofen significantly decreased p-NP concentrations by 41.88% and 27.3%, respectively (*p* < 0.05), while ketoprofen at ¼ MIC significantly decreased p-NP concentrations by 41.62% and 27.1% in *S. aureus* isolates no. 25SA and 47SA, respectively. Ketorolac at ½ MIC significantly decreased p-NP concentrations by 41.19% and 30.99% in *S. aureus* isolates no. 25SA and 47SA, respectively, while at ¼ MIC, ketorolac significantly decreased p-NP concentrations in *S. aureus* isolates no. 25SA and 47SA by 22.83% and 22.73%, respectively. Meloxicam showed significant decrease at ½ and ¼ MIC in isolate no. 47SA by 58.5% for both concentrations. As well, indomethacin at ½ MIC significantly decreased p-NP concentrations in isolates no. 25SA and 47SA by 47.06% and 24.6%, respectively. At ¼ MIC, indomethacin significantly decreased p-NP concentrations by 38.96% in isolates no. 25SA. Similarly, the p-NP concentrations of *S. aureus* isolates no. 25SA and 47SA decreased significantly by 48.6% and 89.07% when these isolates were treated with ½ MIC of celecoxib, respectively. Likewise, celecoxib at ¼ MIC illustrated significant reduction in p-NP concentrations by 48.57% and 78.7% in isolates no. 25SA and 47SA, respectively (Figure 8).

### 2.8. Downregulation of QS and Associated Virulence Factor Genes

The *nuc* gene was used as a housekeeping gene in *S. aureus* isolates 15SA and 47SA, both untreated and treated with diclofenac, meloxicam, and celecoxib. The QS *agrA* gene and related virulence factors genes, *hlb* and *hld*, in *S. aureus* isolates 15SA and 47SA, was tested under both untreated conditions and after treatment with diclofenac, meloxicam, and celecoxib.

**Figure 9:**
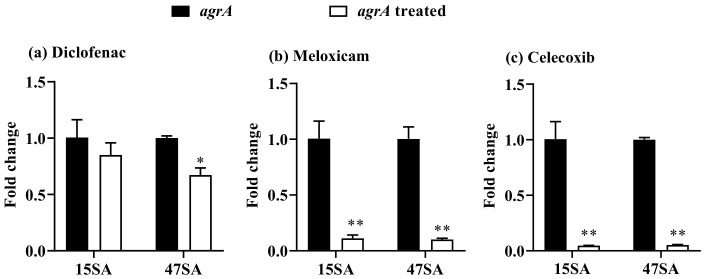
The effect of ½ MIC of (**a**) diclofenac, (**b**) meloxicam, and (**c**) celecoxib on the expression of the QS related gene (*agrA*) in *S. aureus* isolates 15SA and 47SA was evaluated. Statistical analysis was performed using paired *t* test. Data are presented as mean ± SD, with statistical significance indicated at * *p* < 0.05 and ** *p* < 0.01.

Treatment of *S. aureus* isolate 15SA and 47SA with diclofenac resulted in a statistically significant reduction in *agrA* gene expression by 15.5% and 32.7%, respectively (*p* < 0.01). Exposure to meloxicam led to a marked decrease in *agrA*-relative expression in both isolates 15SA and 47SA, with reductions of 88.9% and 89.8%, respectively (*p* < 0.01). Similarly, celecoxib treatment produced a highly significant downregulation of *agrA* expression in isolates 15SA and 47SA, with decreases of 95.3% and 94.7%, respectively (*p* < 0.01) (Figure 9).

**Figure 10:**
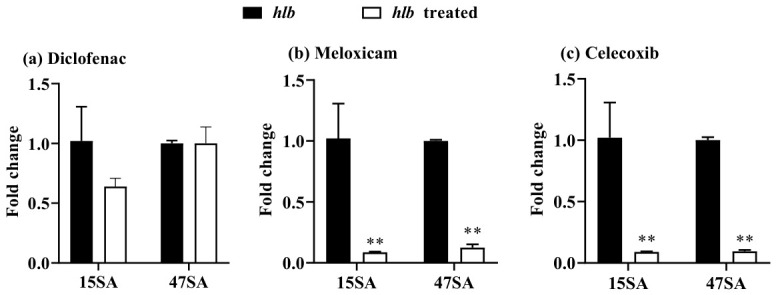
The effect of ½ MIC of (**a**) diclofenac, (**b**) meloxicam, and (**c**) celecoxib on the expression of the QS associated virulence gene (*hlb*) in *S. aureus* isolates 15SA and 47SA was evaluated. Statistical analysis was performed using paired *t* test. Data are presented as mean ± SD, with statistical significance indicated at ** *p* < 0.01.

As shown in Figure 10, treatment of *S. aureus* isolate 15SA with diclofenac resulted in a reduction in *hlb* gene expression by 37.35% (*p* < 0.01). In addition, treatment with meloxicam significantly downregulated *hlb* gene expression in *S. aureus* isolates 15SA and 47SA, resulting in reductions by 91.5% and 87.5%, respectively (*p* < 0.01). Celecoxib exhibited a similarly strong inhibitory effect, with a decrease in *hlb* expression levels by 91.2% in isolate 15SA and 90.5% in isolate 47SA (*p* < 0.01).

**Figure 11:**
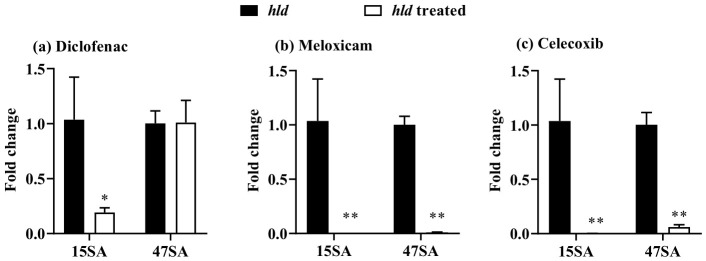
The effect of ½ MIC of (**a**) diclofenac, (**b**) meloxicam, and (**c**) celecoxib on the expression of the QS related virulence factor gene (*hld*) in *S. aureus* isolates 15SA and 47SA was evaluated. Statistical analysis was performed using paired *t* test. Data are presented as mean ± SD, with statistical significance indicated at * *p* < 0.05 and ** *p* < 0.01.

In Figure 11, treatment of *S. aureus* isolate 15SA with diclofenac resulted in a statistically significant reduction in *hld* gene expression by 81.5% (*p* < 0.01). Meloxicam treatment produced a profound downregulation of *hld* expression in both isolates 15SA and 47SA, with reductions of 99.9% and 98.9%, respectively (*p* < 0.01). Similarly, celecoxib demonstrated a strong inhibitory effect, decreasing *hld* expression by 99.8% in isolate 15SA and 93.9% in isolate 47SA (*p* < 0.01).

### 2.9. Molecular Docking of NSAIDs Drugs with AgrA

AutoDock Vina version 1.1.2 (11 May 2011) was used for molecular docking of meloxicam and celecoxib and these results are illustrated in Table 2. Meloxicam had scoring function equals to −7.3 (kcal/moL) and its interactions include H-bonding with arginine (Arg 233) in the chain (A), lysine (Lys 236) in the chain (A) and thymine DNA base in the chain B (DT B3).

**Figure 12:**
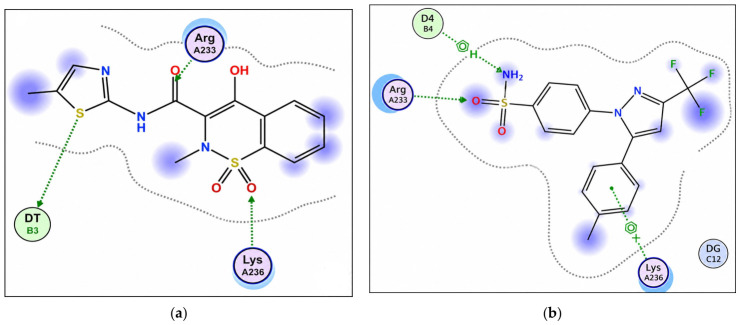
The binding mode and hydrogen bonding interaction of *S. aureus* AgrA (3BS1) with drugs with the highest scores. (**a**) Meloxicam. (**b**) Celecoxib.

Similarly, the scoring function of celecoxib equals to −6.7 (kcal/moL). The interactions of celecoxib were H-bonding with arginine (Arg 233) in the chain (A), arene–cation with lysine (Lys 236) in the chain (A) and arene–H-bonding with adenine DNA base in the chain B (DA B4). These findings indicate that meloxicam and celecoxib exhibited favorable interactions with the AgrA DNA-binding domain, supporting their potential as QS inhibitors targeting AgrA-mediated gene regulation in *S. aureus*. The 2D ligand–target interactions of meloxicam and celecoxib with AgrA are presented in Figure 12.

## 3. Discussion

The Agr QS system is a key global regulator of virulence in *S. aureus*, controlling the expression of multiple exoproteins, including hemolysins, lipases, proteases, PSMs, toxins, and capsular polysaccharides [23]. It also downregulates the expression of cell surface proteins such as protein A and fibronectin-binding proteins [24]. In light of the growing antimicrobial resistance, targeting the QS system in *S. aureus* represents a promising alternative to conventional antibiotics. Quorum sensing quenchers (QSQs) disrupt bacterial communication, thereby attenuating the coordinated expression of virulence factors [25].

In the present study, all *S. aureus* isolates showed complete resistance to cefepime. High cefepime resistance has been reported in Egypt, including 100% resistance among wound-derived MRSA, underscoring the limited efficacy of fourth-generation cephalosporins [26].

The current study also demonstrated high cefoxitin resistance (82.14%) (Supplementary Figure S2), this is consistent with the high MRSA burden reported across Egyptian surveillance studies. Previous surveys reported similarly high MRSA prevalence in clinical isolates; 80% [27] and 67% [28]. Resistance to other antimicrobials varied, with high ceftazidime resistance (91.07%) highlighting the limited efficacy of cephalosporins (Supplementary Figure S2). Similar high resistance rates have been reported: 100% in Egypt [29]. In this study, resistance to amoxicillin/clavulanic acid (21.43%) was is comparable to 50% resistance among *S. aureus* isolates in Cairo, indicating broad β-lactam non-susceptibility [30].

This study showed Macrolide (clarithromycin) resistance in 12.5% of the isolates (Supplementary Figure S2). As well, clindamycin resistance was detected in 3.57% of isolates (Supplementary Figure S2), while other studies reported higher rates, such as 54.5% in Egypt [31]. Resistance to fluoroquinolones (levofloxacin) in this study reported in 3.57% (Supplementary Figure S2). Reported resistance rates vary widely, from 5.7% in China [32], while resistance to tetracyclines (doxycycline) was detected in 12.5% of *S. aureus* isolates (Supplementary Figure S2), compared to 48.8% reported in Egypt [33].

Moreover, 25% of the tested *S. aureus* isolates could be VRSA, and 1.79% exhibited intermediate resistance (VISA) (Figure 3a). This indicates a concerning emergence of reduced vancomycin susceptibility. Several studies in Egypt detected high prevalence of VRSA [34,35,36,37]. In contrast, a meta-analysis in Egypt reported VRSA rate of approximately 9%, reflecting substantial regional variability [28].

Also, susceptibility to linezolid, sulfamethoxazole/trimethoprim and meropenem (Supplementary Figure S2) was detected among the tested isolates. Linezolid susceptibility remains high, consistent with low resistance rates reported before [38], though resistance has been observed (9.7% of 113 isolates in Egypt [39] and 1 of 797 isolates in China [40]) likely reflecting differences in antimicrobial stewardship practices.

The findings of this study reveal marked heterogeneity in antimicrobial resistance profiles among *S. aureus* isolates. These findings highlight the therapeutic challenges of invasive infections and emphasize the need for alternative strategies targeting virulence rather than bacterial survival.

The growing burden of antimicrobial resistance in *S. aureus* cannot be fully understood without considering its close relationship with bacterial virulence. Increasing evidence indicates that resistance and virulence are interconnected through global regulatory networks, particularly the Agr QS system, which coordinates the expression of toxins and other virulence factors [41]. Consequently, multidrug-resistant *S. aureus* strains can retain high pathogenic potential despite extensive antimicrobial resistance [42]. These observations highlight the importance of integrating antimicrobial resistance profiling with virulence assessment and support antivirulence strategies targeting regulatory pathways as complementary approaches to conventional antimicrobial therapy.

The Agr QS system regulates the expression of several extracellular virulence factors, including hemolysins [43], proteases [44], and lipases [45], which collectively contribute to tissue damage, immune evasion, nutrient acquisition, and bacterial persistence [46,47,48]. In the present study, 42.86%, 89.28%, and 94.64% of the clinical isolates produced hemolysin, protease, and lipase, respectively (Figure 4). These findings are generally consistent with previous reports demonstrating the widespread distribution of these Agr-regulated virulence determinants among clinical *S. aureus* isolates, although prevalence varies according to geographical location, infection source, and study methodology [49,50,51,52,53,54]. The frequent co-expression of these virulence factors highlights the pathogenicity of the investigated isolates and supports their selection for subsequent antivirulence evaluation.

In addition, *S. aureus* isolates from various clinical sources showed elevated protease and lipase activities, suggesting roles in tissue invasion and nutrient acquisition. The concurrent detection of hemolysin, protease, and lipase indicates the presence of highly virulent strains capable of causing persistent severe infections and highlights their adaptation for long-term survival in host environments.

The Agr QS system is a major regulator of *S. aureus* virulence, controlling the expression of numerous toxins and extracellular virulence factors [55,56]. The results of this research revealed that isolates no. 25SA and 47SA were the most representative isolates of hemolysin, protease, and lipase enzymes. Also, isolate 15SA was selected on the basis of its strong production of hemolysin, and protease enzymes. Moreover, isolates 15SA and 47SA were characterized as MRSA, while isolate 25SA exhibited both methicillin and vancomycin resistance (MRSA/VRSA). The selected isolates harbored *agrA* together with multiple Agr-regulated virulence genes, including *hla*, *hlb*, *hld*, *spa*, and *psm*, confirming their high virulence potential. In addition, *hub* and *eta* were detected in two isolates, whereas *tst* was identified only in isolate 47SA. These findings support the selection of isolates 15SA, 25SA, and 47SA for subsequent antivirulence and gene expression analyses (Supplementary Table S3).

Nonsteroidal anti-inflammatory drugs (NSAIDs) are widely used as analgesic, antipyretic, and anti-inflammatory effects through inhibition of cyclooxygenase (COX) enzymes involved in prostaglandin synthesis [57]. The usual adult doses of meloxicam were 7.5–15 mg/day [58], and for celecoxib was 100–200 mg once or twice daily [59]. The tested NSAIDs exhibited variable antibacterial activity against the selected *S. aureus* isolates. Meloxicam and indomethacin showing the lowest MIC values, whereas celecoxib and ketoprofen revealed higher concentrations (Table 1). The observed differences in MIC values suggest variable intrinsic antibacterial activity among the tested NSAIDs.

The bacterial growth inhibition, using sub-MIC concentrations (½ MIC), was performed to evaluate QS modulation and virulence attenuation without affecting bacterial viability. Bacterial growth kinetics of treated *S. aureus* isolates 15SA, 25SA and 47SA with diclofenac, ketoprofen, ketorolac, meloxicam and celecoxib did not significantly alter the growth profiles compared with untreated cultures (Figure 5). However, indomethacin showed only a slight delay in exponential growth in some isolates without completely suppressing bacterial proliferation. These findings indicate that the selected sub-MIC concentrations exerted minimal effects on bacterial viability and suitable for assessing antivirulence activity independently of antibacterial effects.

In this study, NSAIDs at sub-MICs showed varying degrees of suppression of virulence factors among the tested isolates. Meloxicam and celecoxib exhibited the most consistent inhibitory activity across the tested virulence factors.

Meloxicam and celecoxib exhibited marked reductions in hemolytic activity across all examined isolates (Figure 6d,f). Diclofenac displayed moderate inhibitory activity, whereas ketoprofen and ketorolac showed only limited effects, mainly against isolate 15SA (Figure 6a–c). As well, indomethacin displayed selective inhibitory activity, showing moderate suppression in isolates 15SA and 25SA but minimal effect in isolate 47SA, highlighting strain-dependent variability in drug responsiveness (Figure 6e). These differences indicate that the antivirulence properties of NSAIDs are not uniform and may depend on their chemical structure to interact with QS system. Several studies have identified compounds that inhibit *S. aureus* hemolysin activity or expression. For example, quinoxalinediones [60], PLNA34, PLNA522 [61], physalin H, physalin B, and isophysalin B [62] have been shown to inhibit α-hemolysin activity.

Also, treatment with sub-MIC concentrations of NSAIDs significantly reduced protease production, although the extent of inhibition varied among the tested compounds and isolates. Meloxicam exhibited the most consistent antiproteolytic activity across all isolates, whereas ketoprofen showed marked inhibition in isolates 25SA and 47SA (Figure 7). These findings indicate that the antivirulence effects of NSAIDs are both drug and strain dependent.

Because protease expression is positively regulated by the Agr QS system [11], the observed reduction in protease activity may be attributed to their ability to modulate Agr-regulated virulence rather than having antibacterial effects. Reduced protease production may limit tissue invasion, nutrient acquisition, and host tissue damage during infection [47]. The greater activity of meloxicam, and to a lesser extent ketoprofen, supports their potential repurposing as adjunctive antivirulence agents against *S. aureus*. The isolate dependent responses observed with several NSAIDs may reflect differences in the genetic background or regulatory activity of the Agr system among the tested isolates, consistent with previous reports describing strain specific variability in antivirulence responses [63,64].

Furthermore, meloxicam and celecoxib demonstrated significant reduction in lipase production. As lipase production is regulated by the Agr QS system, the reduction in enzymatic activity observed in this study suggests indicated that NSAIDs may attenuate Agr-regulated virulence [11]. This interpretation is consistent with previous reports showing that compounds targeting the Agr QS system suppress lipase expression together with other virulence-associated traits in *S. aureus* [65,66].

Collectively, the inhibition of hemolysin, protease, and lipase production observed in the present study supports the antivirulence potential of NSAIDs, particularly meloxicam and celecoxib, against *S. aureus*. These findings suggest that the observed phenotypic effects may be associated with modulation of the Agr QS system rather than causing direct antibacterial effects.

On the gene expression levels, treatment with NSAIDs resulted in marked downregulation of key QS regulatory and virulence-associated genes in *S. aureus*, although the magnitude of inhibition varied among the tested compounds. Diclofenac significantly reduced the expression of *agrA* and was associated with decreased *hlb* and *hld* transcript levels (Figure 9a, Figure 10a and Figure 11a). Meloxicam and celecoxib produced the greatest reductions in *agrA* expression (>85–95%). This was also accompanied by marked suppression of *hld*, which encodes δ-hemolysin and is directly associated with RNAIII, the principal effector of the Agr QS system (Figure 11b,c) [14]. Also, they simultaneously suppressed *hlb*, which is regulated by *agr* dependent mechanism (Figure 10b,c) [67]. Similarly, solonamide B has been reported to reduce the expression of both *agrA* and *hld* in *S. aureus* [68].

Given the central role of the Agr QS system in regulating virulence traits, the coordinated reduction in *agrA*, *hld*, and *hlb* expression observed in this study suggests that selected NSAIDs may modulate Agr-regulated virulence pathways. The consistent pattern of gene downregulation across the tested isolates suggests that NSAIDs treatment is associated with the attenuation of Agr-regulated virulence. Collectively, these findings indicate that meloxicam and celecoxib possess promising antivirulence activity against *S. aureus*.

Our findings agree with those of Elmesseri et al., who demonstrated that diclofenac and meloxicam attenuated MRSA virulence by suppressing staphyloxanthin biosynthesis and downregulating *hla* together with global regulatory genes [69]. In addition, previous studies reported that several NSAIDs altered the expression of regulatory and virulence-associated genes in *S. aureus*, although the affected genes varied among compounds [70]. Furthermore, Okpala and coauthors reported that celecoxib exhibited strong antistaphylococcal activity and, when combined with oxacillin, showed marked synergistic effects against MRSA, substantially reducing oxacillin MIC and potentially resensitizing resistant strains to β-lactam antibiotics [71].

Molecular docking analysis further supports the possibility that meloxicam and celecoxib interact with the AgrA transcriptional regulator, providing a potential explanation for the observed reduction in Agr-regulated virulence. AgrA plays a central role in the Agr QS system by regulating the expression of hemolysins and numerous other virulence-associated factors in *S. aureus*. Its C-terminal LytTR DNA-binding domain is unique to bacteria and has no known human counterpart, making AgrA an attractive target for antivirulence drug development [72]. Meloxicam exhibited a favorable predicted binding affinity (−7.3 kcal/mol), forming hydrogen bonds with Arg233 and Lys236, residues involved in DNA recognition, as well as an additional interaction with thymine (DT3) (Figure 12a and Table 3). Celecoxib also demonstrated favorable predicted binding, forming interactions with Arg233, Lys236, and DA4 through hydrogen bonding and aromatic interactions (Figure 12b and Table 3). The predicted binding affinities of both COX-2 inhibitors were comparable to those reported for previously described AgrA inhibitors, such as savarin [65].

Taken together, the docking results are consistent with the observed phenotypic and gene expression findings and suggest that meloxicam and celecoxib may interact with the AgrA DNA-binding domain.

Although meloxicam, celecoxib, and diclofenac exhibited antivirulent activity against the tested *S. aureus* isolates, these effects were observed at concentrations higher than the systemic peak plasma concentrations [73,74]. Therefore, any antivirulent effects are unlikely to be achieved in vivo with standard systemic dosing. Nevertheless, these findings do not exclude their potential therapeutic application as topical administration may achieve higher local drug concentration minimizing systemic exposure and associated adverse effects.

Moreover, the significant antivirulence activity observed at sub-MICs suggests that meloxicam, celecoxib, and diclofenac may have potential topical antivirulence agents or as adjuncts to conventional antimicrobial therapy for localized *S. aureus* infections, such as wound infections, skin and soft tissue infections, and diabetic foot ulcers [2,3,4]. However, this activity requires confirmation through in vivo studies evaluating topical drug delivery, tissue penetration, pharmacokinetics, safety, and therapeutic efficacy before clinical application can be considered.

## 4. Materials and Methods

### 4.1. Collection, Isolation and Identification of S. aureus from Different Clinical Samples

A total of 56 clinical specimens were collected from patients’ urine, conjunctivitis, blood, nasal swabs, wounds, and diabetic foot infections. These samples were obtained from multiple healthcare institutions using appropriate sampling techniques. Ethical approval for sample collection was granted by the Scientific Research Ethics Committee of Kafr El Sheikh University (code: KFSIRB200-399). All isolates were cultured on mannitol salt agar and incubated at 37 °C for 24 h, producing characteristic golden-yellow colonies. They were subsequently grown in tryptic soy broth (TSB) (Sigma-Aldrich, Taufkirchen, Germany) under the same conditions. Identification of the isolates as *S. aureus* was confirmed through Gram staining, growth on mannitol salt agar, and standard biochemical tests, including catalase and coagulase assays [75,76]. Colonies of *S. aureus* isolates were stored at 4 °C [77], and a single pure colony from each was inoculated into double-strength tryptic soy broth and incubated at 37 °C with shaking at 150 rpm until reaching the exponential phase. For long-term preservation, 500 µL of bacterial culture was mixed with an equal volume of 50% glycerol (El Nasr Co., Cairo, Egypt) and stored at −80 °C [78]. Subcultures were made before starting any experiment to allow cells restoring their viability [79].

### 4.2. Determination of Antibiotic Susceptibility to Different Classes of Antimicrobial Agents

#### 4.2.1. Kirby–Bauer Disk Diffusion Method

The antimicrobial susceptibility testing was assessed using 11 antimicrobial agents representing different antimicrobial classes β-lactams (amoxicillin/clavulanic acid, cefoxitin, ceftazidime, cefepime, meropenem), fluoroquinolones (levofloxacin), lincosamides (clindamycin), folate inhibitors (sulfamethoxazole/trimethoprim), macrolides (clarithromycin), oxazolidinones (linezolid) and tetracyclines (doxycycline). This test was performed using Mueller–Hinton agar (MHA) (Thermo Scientific™ Oxoid™, Basingstoke, UK) by the Kirby–Bauer disk diffusion method, following the criteria of the Clinical and Laboratory Standards Institute (CLSI) [80]. Pure colonies of *S. aureus* were cultured in TSB overnight at 37 °C. The inoculum density was adjusted to 0.5 McF (1.5 × 10^8^ CFU/mL). Lawn culture was performed on MHA plates by using sterile cotton swabs dipped in the diluted culture. Antimicrobial discs were added to the plates and incubated overnight at 37 °C. The diameters of the inhibition zones around each disc were measured, and isolates were classified as resistant, intermediate or susceptible based on CLSI 2021 [80].

#### 4.2.2. Vancomycin Susceptibility Testing

As the disc diffusion is not recommended by CLSI for accurate susceptibility testing of *S. aureus* to vancomycin [80,81]. Vancomycin MIC was determined using the broth microdilution method in sterile 96-well microtiter plates rather than disc diffusion [82,83,84]. Briefly, each well was filled with 100 µL of TSB. Vancomycin (Vancomix, Sigmatec Pharmaceutical Industries, Cairo, Egypt) stock solution (512 µg/mL) was prepared, and 100 µL was added to the first well, followed by two-fold serial dilutions across the plate, then 100 µL was discarded from the last well. Overnight cultures were diluted to a concentration of 5 × 10^6^ CFU/mL and 10 µL of the bacterial suspension was added to each well, resulting in a final inoculum of approximately 5 × 10^5^ CFU/mL wells without antibiotic served as positive growth controls. After incubation at 37 °C for 24 h, bacterial growth was assessed by observing turbidity. The MIC was defined as the lowest concentration of vancomycin that completely inhibited visible growth. Interpretation of the results was based on CLSI criteria [80] for *S. aureus*: susceptible (S) ≤ 2 µg/mL, intermediate (VISA) = 4–8 µg/mL, and resistant (VRSA) ≥ 16 µg/mL [81,85].

### 4.3. Determination of Different Virulence Factors in the Tested Isolates

#### 4.3.1. Hemolysis Assay

Hemolysis activity was evaluated as reported previously [86]. Red blood cells (RBCs) were washed three times with physiological saline, then resuspended in Tris-buffered saline (0.05 mM Tris-HCl (Sigma Aldrich, Hamburg, Germany) and 0.15 mM NaCl (El Nasr Co., Egypt), pH 7.4) and adjusted to a final concentration of 2% (*v*/*v*) at 4 °C. The assay was initiated by combining 100 µL of the RBC suspension with 1 mL of bacterial culture standardized to 0.5 McF, followed by incubation at 37 °C for 24 h in a shaking incubator at 150 rpm. After incubation, samples were centrifuged at 3000 rpm (956× *g*) for 15 min at 4 °C, and hemoglobin release was quantified by measuring absorbance at 540 nm. The red color intensity, measured at 540 nm, served as an indicator of hemolysin release. The percentage of hemolysis was calculated using the formula: % = [(X−B)/(T−B)] × 100, where B represents the negative control (RBCs incubated with TSB); T represents the positive control (complete lysis using 0.1% sodium dodecyl sulfate (SDS) (El Nasr Co., Egypt)); and X corresponds to the tested sample [87]. Each experiment was conducted in quadruplicate. High OD_540nm_ values indicate increased hemoglobin release and, consequently, greater hemolytic activity.

#### 4.3.2. Protease Assay

Using a 5% *w*/*v* skim milk mixture (Miro, Cairo, Egypt), protease activity was quantitatively determined as described by [88], with modifications based on the methods reported by [84,89,90]. *S. aureus* cultures were first grown in TSB at 37 °C with shaking at 150 rpm for 24 h. The inoculum was then adjusted to 1 McF, transferred into 3 mL of fresh TSB and incubated under the same conditions for an additional 48 h. Following incubation, cultures were centrifuged at 10,000 rpm (10,621× *g*) for 10 min at 4 °C to obtain cell-free supernatant. The supernatant was filtered through a 0.45 µm membrane filter (MilliporeSigma, Merck KGaA, Darmstadt, Germany). A volume of 700 µL of the collected supernatant was mixed with an equal volume of 5% *w*/*v* skim milk and incubated at 37 °C for 24 h. Proteolytic activity was assessed by measuring the optical density (OD) at 600 nm [88]. Enzymatic activity was quantified by comparing the absorbance of the culture supernatant to that of a negative control composed of 5% *w*/*v* skim milk and TSB. Low OD_600nm_ values indicate high casein degradation, reflecting high protease activity, relative to the negative control.

#### 4.3.3. Lipase Assay

Lipase activity was assessed via the substrate para-nitrophenyl palmitate (*p*NPP) (Thermo Scientific, Schwerte, Germany) following the method described previously [91]. Overnight cultures of each *S. aureus* isolate were adjusted to 1 McF in 3 mL of TSB, and incubated at 37 °C for 48 h with shaking at 150 rpm. The cultures were then centrifuged at 10.000 rpm (10,621× *g*) for 10 min at 4 °C, and the resulting supernatants were used for lipase determination. The supernatant was filtered through a 0.45 µm membrane filter (MilliporeSigma, Merck KGaA, Darmstadt, Germany). For the assay, 100 µL of solution A, containing 8 mM *p*NPP dissolved in Isopropanol HPLC grade (Sigma Aldrich, Germany), was combined with 900 µL of solution B, composed of 0.005% Triton X-100 (Sigma Aldrich, Germany), 50 mM Tris-HCl (pH 8.0), and 1 mg/mL Gum Arabic (El Nasr Co., Egypt), at a 1:9 ratio. Subsequently, 100 µL of the bacterial culture supernatant was added to 900 µL of the prepared substrate buffer and incubated in the dark at 37 °C for 1 h with shaking at 150 rpm. Lipase activity was quantified by measuring absorbance at 410 nm using a 96-well plate reader (ELISA) (Epoch™ 2 Microplate Reader, BioTek Instruments, Winooski, VT, USA) [92]. A standard calibration curve was generated using para-nitrophenol (Sigma Aldrich, Germany) as the reference compound. Serial two-fold dilutions were prepared in assay buffer and analyzed under the same conditions. The concentration of para-nitrophenol released per h was calculated from the standard curve. The highest OD_410nm_ value corresponded to para-nitrophenol release and, consequently, increased lipase activity [92].

### 4.4. Detection of Quorum Sensing and Associated Virulence Genes in Some Selected Isolates

Three representative *S. aureus* isolates (15SA, 25SA, and 47SA) were selected based on their virulence phenotypes and antimicrobial resistance profiles for subsequent antivirulence assays.

DNA was obtained via a simple boiling method. Three to four fresh colonies were collected, suspended in 70 μL of nuclease free water and incubated at 95 °C for 10 min in a PCR thermal cycler (Multigene^TM^ OptiMax thermal cycler, Labnet International, Edison, NJ, USA). The boiled cells were centrifuged at 13,000 rpm (16,100× *g* using a Sigma 1-14 centrifuge equipped with rotor No. 22091) for 2 min to pellet the cell debris. The supernatant was aspirated and preserved in a −20 freezer [93,94]. The supernatant was filtered through a 0.45 µm membrane filter (MilliporeSigma, Merck KGaA, Darmstadt, Germany).

*S. aureus* isolates 25SA and 47SA, which exhibited hemolysin, protease, and lipase production, were selected for further investigation. In addition, isolate 15SA, characterized by hemolysin and protease production, was also included in the study. PCR analysis using gene specific oligonucleotide primers was performed to detect QS associated gene (*agrA*) as well as several virulence-related genes, including alpha-, beta-, and delta-hemolysins (*hla*, *hlb* and *hld*), staphylococcal protein A (*spa*), lipase (*hub*), phenol-soluble modulins (*psm*), toxic shock syndrome toxin (*tst*) and exfoliative toxin A (*eta*) (Table 3).

PCR amplification was conducted using a thermal cycler (Multigene^TM^ OptiMax thermal cycler, Labnet, USA). The PCR master mix was prepared according to the instructions provided for the Dreamtaq Green PCR master mix (Thermo Fischer). The cycling conditions included an initial denaturation at 95 °C for 2 min, followed by 35 cycles consisting of denaturation at 95 °C for 30 s, annealing for 30 s, extension at 72 °C for 30 s, with a final extension step at 72 °C for 5 min. The amplified PCR products were resolved by electrophoresis on 1.5% (*w*/*v*) agarose gels, stained with ethidium bromide, and visualized under ultraviolet illumination.

**Table 3:** Oligonucleotide primers utilized in conventional PCR and qRT-PCR.

Gene Name	Type	Sequence	Amplified Product (bp)	Annealing	Reference
*agrA*	Fw Rv	TTAGAAACTGCACATACACGC ATGGGCAATGAGTCTGTGAG	153 bp	54 °C	This study
*hlb*	Fw Rv	CCAAACACCTGTACTCGGTC CATTGTCGAATCCACAACC	162 bp	54 °C	This study
*hla*	Fw Rv	ATGAATCCTGTCGCTAATGC GCAATGGTACCTTTCGTTCT	207 bp	56 °C	This study
*Spa*	Fw Rv	TAAAGACGATCCTTCAGTGAG TGTCTTCCTCTTTTGGTGC	83 bp	53 °C	This study
*hld*	Fw Rv	AGGAAGGAGTGATTTCAATGG GTGAATTTGTTCACTGTGTCGA	88 bp	56 °C	This study
*hub*	Fw Rv	CTTTGAATGCTGGAACTTTAC ATTGGTTTCGGTAACTTTGA	109 bp	52 °C	This study
*Psm*	Fw Rv	TATCAAAAGCTTAATCGAACAATTC CCCCTTCAAATAAGATGTTCATATC	175 bp	57 °C	[61]
*tst*	Fw Rv	GTAAGCCCTTTGTTGCTTGC CTGATGCTGCCATCTGTGTT	215 bp	57 °C	[95]
*eta*	Fw Rv	GTTCCGGGAAATTCTGGATC GCTTGACATAATTCCCAATACC	133 bp	56 °C	This study
*nuc*	Fw Rv	GCGATTGATGGTGATACGGTT AGCCAAGCCTTGACGAACTAAAGC	267 bp	55 °C	[96]

Fw: Forward, Rv: Reverse, bp: Base pair.

### 4.5. Determination of MIC of NSAIDs by Broth Microdilution Method

The antibacterial activity of the tested NSAIDs against *S. aureus* isolates (15SA, 25SA, and 47SA) was evaluated by the broth microdilution method as previously described [80]. Briefly, celecoxib (kindly supplied by Amoun pharmaceutical, Al Obour, Egypt), diclofenac (Sandoz, Cairo, Egypt), ketoprofen (Sanofi Aventis, Cairo, Egypt), ketorolac (Amriya, Alexandria, Egypt), meloxicam (Global pharmaceutical company, Cairo, Egypt), and indomethacin (Nile Co. for pharmaceuticals, Cairo, Egypt) stock suspensions were prepared at concentrations of 25, 25, 50, 15, 10, and 25 mg/mL, respectively. Two-fold serial dilutions of each NSAID were prepared in Mueller–Hinton broth (MHB) (Thermo Scientific™, Oxoid™, Basingstoke, UK) in sterile 96-well microplates (Griener bione, No E20013CP, Frickenhausen, Germany). After incubation at 37 °C for 24 h, bacterial growth was assessed using 0.5% (*w*/*v*) (2, 3, 5-triphenyl tetrazolium; Fischer Chemical, Fair Lawn, NJ, USA) and the MIC was defined as the lowest concentration that completely inhibited visible bacterial growth [97].

### 4.6. The Effect of Sub-MIC on Bacterial Growth

Diclofenac, ketoprofen, ketorolac, meloxicam, indomethacin and celecoxib were evaluated of the effect of sub-MIC concentrations on bacterial growth. A comparative analysis was performed to assess the influence of NSAIDs at (½ MIC) on *S. aureus* isolates (15SA, 25SA and 47SA). Growth kinetics of untreated cultures were compared with those exposed to subinhibitory concentrations of NSAIDs (diclofenac, ketoprofen, ketorolac, meloxicam, indomethacin and celecoxib). Tryptic soy broth (TSB) containing 20% DMSO (Sigma Aldrich, Germany), either supplemented with the selected compounds (½ MIC) or left untreated, was inoculated with overnight *S. aureus* cultures adjusted to the required OD_600nm_. The cultures were incubated at 37 °C for 24 h, and bacterial growth was monitored over time. At predetermined intervals (2, 3, 3.5, 4, 6, 8, 10, and 12 h), aliquots were collected and optical density measurements at 600 nm were recorded for both treated and untreated samples to evaluate growth rate changes [98].

### 4.7. Inhibitory Impact of the Selected Drugs on Key Virulence Factors in S. aureus

To investigate the influence of the tested compounds on virulence expression, overnight cultures of *S. aureus* isolates 15SA, 25SA, and 47SA were cultured in TSB supplemented with sub-MIC levels (¼ and ½ MIC) of NSAIDs. Untreated cultures lacking the tested compounds were prepared under identical conditions to provide a control for comparative analysis of the effect of the added drugs on virulence-related QS.

#### 4.7.1. Hemolysis Assay

The effect of subinhibitory concentrations (¼ and ½ MIC) of NSAIDs on hemolytic activity was assessed using the hemolysis assay described above [methods Section 4.3.1], with minor modifications [86]. Briefly, overnight cultures adjusted to 0.5 McF were exposed to the indicated NSAID concentrations and incubated with red blood cells. Untreated control samples were prepared in parallel by mixing RBCs with overnight bacterial cultures lacking NSAIDs. After incubation, hemoglobin release was quantified by measuring the absorbance at 540 nm. The degree of hemolysis in both treated and untreated cultures was calculated according to the method of Rossignol et al. [87]. For comparative analysis, the hemolytic activity of treated samples was normalized to the corresponding untreated culture, which was defined as 100% hemolytic activity. Hemolytic activity (%) was calculated by expressing the activity of each treated sample as a percentage of its corresponding untreated control: Hemolysin activity (%) = (T/X) × 100, where *T* represents the hemolysis% of the treated sample, and *X* represents the hemolysis% of the corresponding untreated culture. All experiments were carried out in quadruplicate.

#### 4.7.2. Protease Assay

Protease production was determined under treatment with sub-MIC levels (¼ and ½ MIC) of NSAIDs using 5% skim milk assay technique [88]. Cell-free supernatants obtained from treated and untreated cultures were analyzed, and proteolytic activity was determined spectrophotometrically by measuring the absorbance at 600 nm. All measurements were performed in comparison with the corresponding untreated controls. The protease activity of the untreated culture was set to 100% and the protease activity of treated samples was determined using the following equation: Protease activity (%) = (B − T)/(B − X) × 100, where *B* represents the absorbance (OD) of the negative control, *T* represents the absorbance (OD) of the treated sample, and *X* represents the absorbance (OD) of the corresponding untreated culture.

#### 4.7.3. Lipase Assay

Lipase activity was measured quantitatively following the protocol of Gupta and coauthors [91] to determine the effect of subinhibitory concentrations (¼ and ½ MICs) of NSAIDs on *S. aureus* isolates. Lipase activity was quantified spectrophotometrically at 410 nm based on the release of para -nitrophenol using a standard calibration curve. All measurements were performed using cell-free supernatants from treated and untreated cultures. A decrease in OD_410nm_ in treated samples compared with untreated controls indicated reduced enzymatic hydrolysis, demonstrating suppression of bacterial lipase activity by the tested compounds.

### 4.8. Gene Expression Analysis by Quantitative Real-Time Polymerase Chain Reaction (qRT-PCR)

#### 4.8.1. RNA Extraction (Using Lysozyme and TRI Reagent^®^)

The effects of diclofenac, meloxicam, and celecoxib on the expression of the QS gene (*agrA*) and virulence genes (*hlb* and *hld*) in *S. aureus* were evaluated by qRT-PCR. Total RNA was extracted from treated and untreated cultures using TRI reagent^®^ (Sigma Aldrich, St. Louis, MI, USA). After overnight growth in TSB, isolates 15SA and 47SA were exposed to ½ MIC of the tested compounds for 3 h at 37 °C with shaking (150 rpm) until reaching exponential phase (OD_600nm_ = 0.8). Bacterial cells were harvested by centrifugation, resuspended in Tris-EDTA buffer containing lysozyme (10 mg/mL), and incubated at 37 °C for 1 h [99]. The pellets were then mixed with TRI reagent and stored at −80 °C for RNA extraction.

The samples underwent three freeze–thaw cycles, followed by incubation at room temperature for 5 min to achieve complete dissociation of nucleoprotein complexes. Chloroform (100 µL) was then added, and the mixtures were vortexed for 15 s, incubated for 2–3 min at room temperature, and centrifuged at 12,000 rpm (15,294× *g*) for 15 min at 4 °C using a refrigerated centrifuge (Sigma 2-16KL, Germany). This process produced three layers: an upper aqueous phase containing RNA, an interphase containing DNA, and a lower phenol-chloroform layer. The aqueous phase was carefully transferred to RNase-free tubes, and chloroform extraction was repeated twice to maximize RNA recovery.

RNA precipitation was achieved by adding 250 µL of 100% isopropanol to the aqueous phase, followed by incubation at room temperature for 5 min and centrifugation at 12,000 rpm (15,294× *g*) for 10 min at 4 °C. The resulting RNA pellet was washed twice with 75% ethanol prepared in DEPC-treated water and centrifuged at 10,000 rpm (10,621× *g*) for 5 min at 4 °C. After removal of the supernatant, the pellet was air-dried for 10 to 15 min and dissolved in 20 µL DEPC-treated water (Thermo Scientific™, Waltham, MA, USA). The RNA samples were incubated at 65 °C for 30 min for stabilization and then stored at −80 °C. RNA concentration and purity were determined spectrophotometrically using a NanoPhotometer^®^ P330 (IMPLEN GmbH, Munich, Germany) by measuring absorbance at 260 nm and the 260/280 nm ratio, respectively.

#### 4.8.2. qRT-PCR Assay

Gene amplification and expression analysis were carried out using Trans Script^®^ Green One-Step qRT-PCR Super Mix according to the manufacturer’s protocol. qRT-PCR reactions were performed on a Rotor-Gene Q thermocycler (StepOne^®^ Real-Time PCR system, Life Technologies^TM^, Singapore). The cycling conditions included cDNA synthesis at 45 °C for 7 min, initial denaturation at 94 °C for 1 min, followed by 45 cycles of denaturation at 94 °C for 15 s, annealing for 30 s at the temperatures listed in Table 3, and extension at 72 °C for 30 s. Expression levels were normalized against the *nuc* housekeeping gene of *S. aureus*. All reactions were performed in duplicate to ensure reproducibility. Relative changes in gene expression were calculated from Ct values using the ΔΔCt method, and results were expressed as 2^−ΔΔCt^ for treated samples relative to untreated controls [100].

### 4.9. Molecular Docking

Meloxicam and celecoxib were obtained as SDF files (3D conformer) from PubChem (https://pubchem.ncbi.nlm.nih.gov/) (accessed on 9 May 2026), then prepared for docking based virtual screening. The database was converted into Molecular Data Base (MDB) files by Open Bapel GUI (version 3.1.1) [101]. Next, the PDB files were converted into PDB-qt files using autoDockTools-1.5.7rc1 [102].

*S. aureus* AgrA X-ray crystallographic structure (PDB ID: 3BS1) was retrieved from the PDB website (https://www.rcsb.org/). Prior to docking analysis, all water molecules were removed using AutoDockTools-1.5.7rc1 (ADT), Kollman charges were then applied, hydrogen polarities along with polar hydrogen atoms were assigned. Subsequently, the protein structure file was converted from PDB to PDBQT format. The targeted binding region corresponded to the groove located between Val232 and Lys236 and included the amino acid residues His200, Asn201, Arg218, Tyr229, Ser231, Val232 and Lys237 [72,103].

Molecular docking analysis was subsequently carried out using AutoDock Vina (version 1.1.2) [102], where docking calculations were executed automatically. The generated binding poses were then analyzed, and those exhibiting the most favorable ligand–protein interactions were selected [104].

### 4.10. Statistical Analysis

Mean values, standard deviations (SDs), and para-nitrophenol release rates were calculated using Microsoft Excel 2016, while statistical analyses were performed with GraphPad Prism version 8.0.2.263. Differences between treated and untreated groups were analyzed using one-way ANOVA followed by Dunnett’s multiple comparison test, and paired *t* test where appropriate. Growth curves were analyzed using two-way repeated-measures ANOVA followed by Sidac’s multiple comparisons test. Differences were considered statistically significant at *p* < 0.05. All experiments were performed in quadruplicate or sextuplicate. Statistical significance was considered at *p* < 0.05, whereas *p* < 0.01 indicated high significance.

## 5. Conclusions

The present study illustrates the vital role of the Agr QS system in regulating the expression of multiple virulence factors in *S. aureus*, including hemolysins, proteases, and lipases, which collectively drive pathogenicity. The high prevalence of MRSA and the emergence of VRSA among clinical isolates highlight the growing threat of multidrug-resistant *S. aureus* and the limitations of conventional antibiotic therapy. Importantly, the results demonstrate that selected NSAIDs can be repurposed as potent QS inhibitors, effectively attenuating virulence without compromising bacterial growth, thereby minimizing selective pressure for resistance development. Among these, meloxicam, and celecoxib exhibited the strongest and broadest antivirulence activity, consistently suppressing hemolysin, protease, and lipase production. Molecular docking and qRT-PCR analysis confirmed that meloxicam and celecoxib, may modulate the Agr QS pathway, leading to marked downregulation of *agrA* and its downstream targets *hlb* and *hld*, thereby silencing QS mediated virulence programs. Collectively, these findings establish that meloxicam and celecoxib were associated with reduced activity of the QS via drug repurposing as a promising antivirulence strategy to combat MRSA and VRSA infections, offering a complementary therapeutic approach to conventional antibiotics and a potential means to combat virulence in multidrug-resistant *S. aureus* particularly those with inflammation.

## Figures and Tables

**Table 1:** MICs of the selected NSAIDs against *S. aureus* isolates no. 15SA, 25SA and 47SA.

Isolate no.	15SA	25SA	47SA
Drugs	MIC (mg/mL)	MIC (mg/mL)	MIC (mg/mL)
	0.156	2.5	0.3125
ketoprofen	2.5	5	2.5
Ketorolac	1.25	2.5	2.5
Meloxicam	0.625	2.5	1.25
Indomethacin	0.312	2.5	0.312
Celecoxib	5	1.25	5

no: number, MIC: minimal inhibitory concentration.

**Table 2:** Docking scores of drugs with the highest results with *S. aureus* AgrA via MOE.

Target Protein	Drug Name	Docking Score (kcaL/moL)	The Interaction with *S. aureus* AgrA and DNA
AgrA (3bs1)	Meloxicam	−7.3	H-bonding with argenine (Arg 233) in the chain (A), lysine (Lys 236) in the chain (A) and thymine base in DNA *B*3 (DT B3).
	Celecoxib	−6.7	H-bonding with argenine (Arg 233) in the chain (A), lysine (Lys 236) in the chain (A) and thymine base in DNA *B*3 (DT B3).

## Data Availability

All data developed or analyzed during the current study are provided in the manuscript. The Supplementary Information Files are available from the corresponding author upon reasonable request.

## Supplementary Materials

The following supporting information can be downloaded at: https://www.mdpi.com/article/10.3390/antibiotics15070707/s1, Supplementary Table S1: Sources of *S. aureus* isolates; Supplementary Table S2: Determination of MRSA, MSSA, VISA and VRSA among *S. aureus* isolates; Supplementary Table S3. Quorum sensing and associated virulence genes in some selected *S. aureus* isolates; Supplementary Figure S1: Representative Gram stained microscopic image of *S. aureus* clinical isolates; Supplementary Figure S2: Distribution of resistance of antimicrobial agents among different clinical sources of *S. aureus* (urine, conjunctivitis, blood, nose, wound, diabetic foot). AMC: amoxicillin/clavulanic acid, CX: cefoxitin, CAZ: ceftazidime, FEB: cefepime, MEM: meropenem, LE: levofloxacin, CMN: clindamycin, SXT: sulfamethoxazole/trimethoprim, CLR: clarithromycin, LZD: linezolid, DO: doxycycline.

## Abbreviations

The following abbreviations are used in this manuscript:

*S. aureus*	*Staphylococcus aureus*
*agr*	accessory gene regulator
QS	quorum sensing
NSAIDs	nonsteroidal anti-inflammatory drugs
TSB	tryptic soy broth
QQ	Quorum Quenching
QSI	quorum sensing inhibitor
MIC	minimum inhibitory concentration
Sub-MIC	subinhibitory concentration
OD	optical density
qRT-PCR	quantitative real-time polymerase chain reaction
Ct	cycle threshold
*hla*	hemolysins α
*hlb*	hemolysins β
*hld*	hemolysins δ
*agrA*	accessory gene regulator A (response regulator)
*psm*	phenol-soluble modulin
*eta*	exfoliative toxin A
*tst*	toxic shock syndrome toxin
*spa*	surface protein A
