# Supplementary material for: Suppression of Quorum Sensing and Virulence Factors by Meloxicam and Celecoxib in Methicillin-Resistant Staphylococcus aureus Clinical Isolates
Source: Antibiotics (Basel). 2026 Jul 21;15(7):707. doi: 10.3390/antibiotics15070707 (PMC13405825; doi:10.3390/antibiotics15070707)
Supplement: Supplementary file 1 [file antibiotics-15-00707-s001.zip › antibiotics-4402892-supplementary.pdf]

antibiotics

MDPI

Article

Suppression of *Staphylococcus aureus* Quorum Sensing and Virulence Factors by Meloxicam and Celecoxib

Reham Ali [1], Ramadan A. El-Domany [1] and Mona I. Shaaban [2,*]

[1] Department of Microbiology and Immunology, Faculty of Pharmacy Kafrelsheikh University, Kafr El Sheikh 33516, Egypt; reham_mansour@pharm.kfs.edu.eg
[2] Department of Microbiology and Immunology, Faculty of Pharmacy, Mansoura University, Mansoura 35516, Egypt; mona_ibrahem@mans.edu.eg
* Mona I. Shaaban
Mail; mona_ibrahem@mans.edu.eg

Supplementary Materials

Table S1. Sources of *S. aureus* isolates.

Isolate code	Source	Isolate code	Source
1 SA	Urine sample	29 SA	Nose swap
2 SA	Urine sample	30 SA	Nose swap
3 SA	Urine sample	31 SA	Nose swap
4 SA	Urine sample	32 SA	Nose swap
5 SA	Urine sample	33 SA	Nose swap
6 SA	Urine sample	34 SA	Nose swap
7 SA	Urine sample	35 SA	Lower limb amputation
8 SA	Urine sample	36 SA	Abscess
9 SA	Urine sample	37 SA	Foot gangrene
10 SA	Urine sample	38 SA	Lower limb amputation
11 SA	Conjunctivitis	39 SA	Pus swap from wound
12 SA	Conjunctivitis	40 SA	Pus swap form acne
13 SA	Conjunctivitis	41 SA	Lower limb amputation
14 SA	Conjunctivitis	42 SA	Venous ulcer lower limb
15 SA	Conjunctivitis	43 SA	Hand infection
16 SA	Conjunctivitis	44 SA	Hand abscess
17 SA	Conjunctivitis	45 SA	Wound
18 SA	Conjunctivitis	46 SA	Wound
19 SA	Conjunctivitis	47 SA	Diabetic foot
20 SA	Conjunctivitis	48 SA	Diabetic foot
21 SA	Blood sample	49 SA	Diabetic foot
22 SA	Blood sample	50 SA	Diabetic foot
23 SA	Blood sample	51 SA	Diabetic foot
24 SA	Blood sample	52 SA	Diabetic foot
25 SA	Nose swap	53 SA	Diabetic foot
26 SA	Nose swap	54 SA	Diabetic foot

 https://doi.org/10.3390/antibiotics15070707

27 SA	Nose swap	55 SA	Diabetic foot
28 SA	Nose swap	56 SA	Diabetic foot

SA: *Staphylococcus aureus*.

Table S2. Determination of MRSA, MSSA, VISA and VRSA among *S. aureus* isolates

Isolate code	Susceptibility to Cefoxitin	MRSA/ MSSA	MIC to Vancomycin (µg/mL)	VISA/VRSA	Hemolysin (hemolysis %±SD)	Protease (OD±SD)	Lipase (conc. (mmol) of PNP/ h±SD)
1 SA	R	MRSA	< 2	S	92.96±0.1	1.52±0.19	0.08±0
2 SA	R	MRSA	256	R (VRSA)	23.23±0.05	1.68±0.17	0.99±0.02
3 SA	S	MSSA	< 2	S	6.49±0.02	1.57±0.06	0.99±0.02
4 SA	R	MRSA	< 2	S	37.26±0.01	1.75±0.02	0.16±0.02
5 SA	R	MRSA	32	R (VRSA)	91.80±0.01	1.78±0.02	0.47±0.03
6 SA	R	MRSA	4	I (VISA)	17.47±0.01	1.64±0.24	0.93±0.04
7 SA	S	MSSA	< 2	S	13.59±0.01	1.34±0.004	0.74±0.05
8 SA	R	MRSA	< 2	S	63.41±0.19	1.91±0.19	1±0
9 SA	R	MRSA	64	R (VRSA)	3.71±0.01	2.09±0.06	0.97±0.02
10 SA	S	MSSA	< 2	S	10.63±0.01	0.94±0.2	0.97±0.03
11 SA	R	MRSA	< 2	S	6.70±0.01	1.67±0.15	0.95±0.04
12 SA	R	MRSA	64	R (VRSA)	18.41±0.1	1.9±0.02	0.81±0.02
13 SA	S	MSSA	64	R (VRSA)	95.36±0.02	1.79±0.01	0.59±0.03
14 SA	R	MRSA	< 2	S	10.69±0.03	2.01±0.17	0.93±0.02
15 SA	R	MRSA	< 2	S	98.66±0.04	1.38±0.05	0.87±0.05
16 SA	R	MRSA	< 2	S	7.17±0.03	1.71±0.01	0.17±0.03
17 SA	R	MRSA	< 2	S	1.08±0.01	1.39±0.24	0.22±0.02
18 SA	R	MRSA	< 2	S	1.67±0.02	1.06±0.02	0.35±0.03
19 SA	R	MRSA	< 2	S	46.85±0.13	1.86±0.11	0.33±0.09
20 SA	R	MRSA	< 2	S	98.66±0.02	1.65±0.23	0.9±0.12
21 SA	R	MRSA	64	R (VRSA)	8.99±0.02	1.33±0.17	0.65±0.03
22 SA	R	MRSA	< 2	S	3.03±0.03	1.95±0.06	0.47±0.1
23 SA	R	MRSA	< 2	S	1.61±0.002	1.35±0.14	0.44±0.07
24 SA	R	MRSA	< 2	S	95.96±0.15	1.29±0.01	0.44±0.12
25 SA	R	MRSA	256	R (VRSA)	53.56±0.01	0.33±0.19	0.46±0.02
26 SA	R	MRSA	< 2	S	11.73±0.1	1.64±0.04	0.77±0.15
27 SA	R	MRSA	< 2	S	13.99±0.02	1.85±0.01	0.91±0.02
28 SA	R	MRSA	< 2	S	24.91±0.03	0.34±0.08	0.37±0.08
29 SA	R	MRSA	< 2	S	6.83±0.04	1.89±0.15	0.96±0.03
30 SA	R	MRSA	< 2	S	2.61±0.01	1.76±0.31	0.64±0.02
31 SA	R	MRSA	< 2	S	2.39±0.01	1.69±0.03	0.97±0.02
32 SA	R	MRSA	256	R (VRSA)	9.29±0.05	1.76±0.01	0.62±0
33 SA	R	MRSA	< 2	S	10.56±0.01	1.74±0.02	0.97±0.03
34 SA	R	MRSA	< 2	S	29.78±0.01	1.57±0.09	0.82±0.17
35 SA	R	MRSA	< 2	S	23.63±0.097	1.81±0.01	0.64±0.01
36 SA	R	MRSA	< 2	S	90.43±0.18	1.73±0.06	0.59±0.11
37 SA	R	MRSA	256	R (VRSA)	17.84±0.1	1.6±0.06	0.28±0.04
38 SA	S	MSSA	< 2	S	10.87±0.03	1.78±0.09	0.1±0.01
39 SA	R	MRSA	< 2	S	13.68±0.06	2.04±0.02	0.75±0.06
40 SA	R	MRSA	< 2	S	70.16±0.14	1.77±0.04	0.92±0.01
41 SA	R	MRSA	256	R (VRSA)	2.38±0.01	1.45±0.3	0.94±0.02
42 SA	R	MRSA	< 2	S	0.71±0.01	1.68±0.05	0.83±0.08
43 SA	S	MSSA	< 2	S	7.04±0.01	1.85±0.05	0.13±0.02
44 SA	R	MRSA	< 2	S	17.46±0.07	1.76±0.04	0.58±0.07

https://doi.org/10.3390/antibiotics15070707

45 SA	S	MSSA	< 2	S	10.19±0.01	1.9±0.17	0.96±0.01
46 SA	S	MSSA	< 2	S	7.55±0.02	1.87±0.19	0.35±0.07
47 SA	R	MRSA	< 2	S	98.50±0.02	1.32±0.1	0.91±0.01
48 SA	R	MRSA	32	R (VRSA)	5.79±0.01	1.45±0.06	0.15±0
49 SA	R	MRSA	< 2	S	54.69±0.24	1.83±0.13	0.9±0.09
50 SA	R	MRSA	< 2	S	6.62±0.01	0.76±0.06	0.35±0.03
51 SA	R	MRSA	< 2	S	10.54±0.01	1.89±0.07	0.99±0.02
52 SA	R	MRSA	< 2	S	18.23±0.05	1.89±0.02	0.62±0.07
53 SA	S	MSSA	256	R (VRSA)	27.31±0.09	0.54±0.13	0.41±0
54 SA	R	MRSA	< 2	S	44.06±0.11	1.84±0.18	0.73±0.02
55 SA	S	MSSA	256	R (VRSA)	1.23±0.01	2.11±0.09	0.94±0.08
56 SA	R	MRSA	128	R (VRSA)	11.06±0.07	1.8±0.04	0.37±0.05

MRSA: methicillin resistant *S. aureus*, MSSA: methicillin sensitive *S. aureus*, VISA: vancomycin intermediate *S. aureus*, and VRSA: vancomycin resistant *S. aureus*.

R: resistant, I: intermediate, S: sensitive.

Table S3. Quorum sensing and associated virulence genes in some selected *S. aureus* isolates.

Isolates	*agrA*	*hla*	*hlb*	*hld*	*spa*	*psm*	*tst*	*hub*	*eta*
**15SA**	+	+	+	+	+	+	-	-	-
**25SA**	+	+	+	+	+	+	-	+	+
**47SA**	+	+	+	+	+	+	+	+	+

(+): positive; (-): negative

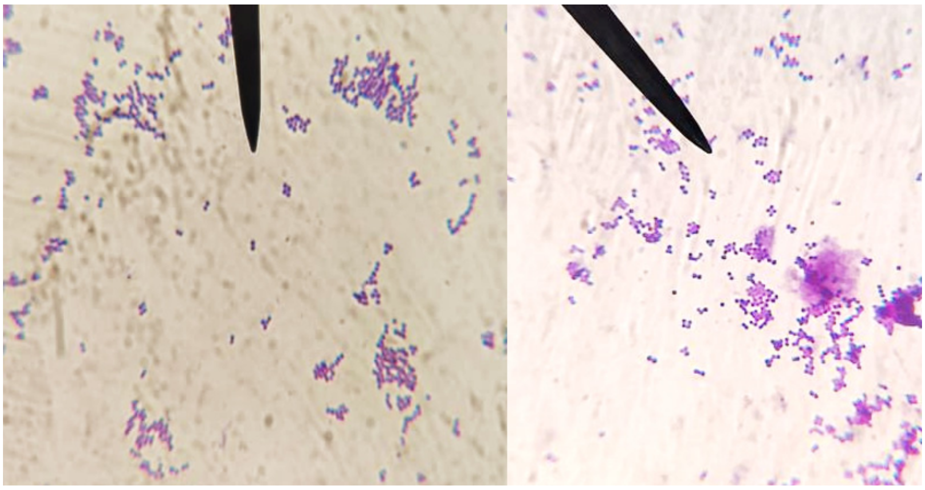

Figure S1. Representative Gram stained microscopic image of *S. aureus* clinical isolates.

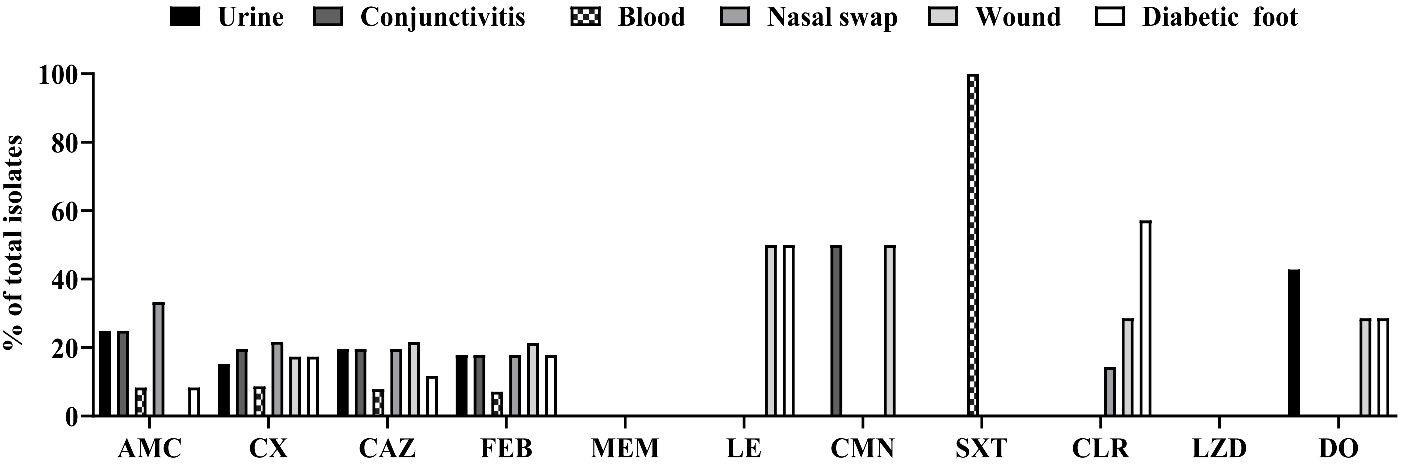

Figure S2. Distribution of resistance of antimicrobial agents among different clinical sources of *S. aureus* (urine, conjunctivitis, blood, nose, wound, diabetic foot). **AMC:** amoxicillin/clavulanic acid, **CX:** cefoxitin, **CAZ:** ceftazidime, **FEB:** cefepime, **MEM:** meropenem, **LE:** levofloxacin, **CMN:** clindamycin, **SXT:** sulfamethoxazole/trimethoprim, **CLR:** clarithromycin, **LZD:** linezolid, **DO:** doxycycline.

https://doi.org/10.3390/antibiotics15070707
